# Patient Priorities Concerning Treatment Decisions for Advanced Neuroendocrine Tumors Identified by Discrete Choice Experiments

**DOI:** 10.1093/oncolo/oyad312

**Published:** 2023-11-25

**Authors:** Matthew Anaka, David Chan, Sharon Pattison, Alia Thawer, Bryan Franco, Lesley Moody, Christopher Jackson, Eva Segelov, Simron Singh

**Affiliations:** Cross Cancer Institute, Alberta Health Services, Edmonton, Alberta, Canada; Department of Medicine, University of Alberta, Edmonton, Alberta, Canada; Northern Sydney Cancer Centre, St Leonards, New South Wales, Australia; Department of Medicine, Otago Medical School, University of Otago, Dunedin, Otago, New Zealand; Odette Cancer Centre, Sunnybrook Health Sciences Centre, Toronto, Ontario, Canada; Department of Medicine, University of Alberta, Edmonton, Alberta, Canada; Princess Margaret Cancer Centre, Toronto, Ontario, Canada; Department of Medicine, Otago Medical School, University of Otago, Dunedin, Otago, New Zealand; Department of Clinical Research, University of Bern, Bern, Switzerland; Department of Medicine, Monash University, Melbourne, Victoria, Australia; Department of Medicine, University of Toronto, Toronto, Ontario, Canada

**Keywords:** neuroendocrine tumors, decision making, patient preferences, antineoplastic protocols

## Abstract

**Background:**

Patients with advanced neuroendocrine tumors (NETs) have multiple treatment options. Ideally, treatment decisions are shared between physician and patient; however, previous studies suggest that oncologists and patients place different value on treatment attributes such as adverse event (AE) rates. High-quality information on NET patient treatment preferences may facilitate patient-centered decision making by helping clinicians understand patient priorities.

**Methods:**

This study used 2 discrete choice experiments (DCE) to elicit preferences of NET patients regarding advanced midgut and pancreatic NET (pNET) treatments. The DCEs used the “potentially all pairwise rankings of all possible alternatives” (PAPRIKA) method. The primary objective was to determine relative utility rankings for treatment attributes, including progression-free survival (PFS), treatment modality, and AE rates. Ranking of attribute profiles matching specific treatments was also determined. Levels for treatment attributes were obtained from randomized clinical trial data of NET treatments.

**Results:**

One hundred and 10 participants completed the midgut NET DCE, and 132 completed the pNET DCE. Longer PFS was the highest ranked treatment attribute in 64.5% of participants in the midgut NET DCE, and in 59% in the pNET DCE. Approximately, 40% of participants in both scenarios prioritized lower AE rates or less invasive treatment modalities over PFS. Ranking of treatment profiles in the midgut NET scenario identified 60.9% of participants favoring peptide receptor radionuclide therapy (PRRT), and 30.0% somatostatin analogue dose escalation.

**Conclusion:**

NET patients have heterogeneous priorities when choosing between treatment options based on the results of 2 independent DCEs. These results highlight the importance of shared decision making for NET patients.

## Teaser

Treatment decisions are shared between physician and patient; however, studies suggest that oncologists and patients place different value on treatment attributes such as adverse event rates. High-quality information on neuroendocrine tumor treatment preferences may facilitate patient-centered decision making by helping clinicians understand patient priorities.

**Implications for Practice**

This study investigated neuroendocrine tumor (NET) patient treatment preferences using an adaptive online research platform where participants chose between hypothetical treatments that differed based on characteristics like associated length of time without tumor growth, side effects profiles, and how treatments are given. We found that while most patients placed priority on longer duration of cancer control, many patients would prioritize less side effects or less invasive treatments over longer cancer control. This information highlights that not all NET patients will prefer the same treatments, and that this decision should be shared between a physician and their patients.

## Introduction

Neuroendocrine tumors (NETs) are a diverse group of malignancies, with significant heterogeneity in prognosis, symptom burden, and impact on quality of life. Due to the uncommon nature of this disease, head-to-head clinical trials are lacking, with limited data available to guide the sequence of treatments. As such shared decision making between patients and physicians is critical, and involves eliciting and understanding patient preferences.^1^ Despite recent efforts to catalogue the challenges faced by patients with a NET,^2^ there is a relative scarcity of information on NET patient preferences and priorities for available treatments. Provision of patient-centered care requires an understanding of patient values and preferences.^3,4^ However existing data suggests that oncologists and patients place different value on treatment attributes such as risk of adverse events and the treatment modality.^5-7^ This may hinder efforts to provide patient-centered care, as physicians may introduce bias during information sharing even while aiming to participate in shared decision making.^8,9^ High-quality information on NET patient preferences regarding available treatment options has the potential to facilitate patient-centered, shared decision making by giving physicians a priori information on the nature of patient preferences and priorities. Furthermore, clearly defined patient priorities can help guide regulatory decision making, value-based pharmacoeconomic decision making, and clinic trial design through inclusion of patient-centered outcome measures.

There are multiple treatment options for advanced, unresectable midgut NETs and pancreatic NETs (pNETs). Asymptomatic patients with low tumor burden at diagnosis can either be followed with observation, or treated with long-acting somatostatin analogues to slow tumor growth.^10^ Patients with a higher tumor burden or a functional tumor at diagnosis are typically initially treated with somatostatin analogues (SSAs).^11,12^ After radiologic, biochemical, or symptomatic progression on SSAs, options for advanced midgut NETs include SSA dose escalation,^13^ targeted small molecule drugs such as everolimus,^14^ or peptide receptor radionuclide therapy (PRRT).^15^ The approach for pNETs is similar. Treatment options for progressive, symptomatic, or clinically significant tumor burden include SSAs,^11^ and targeted agents including everolimus^16^ and sunitinib.^17^ PNETS are more responsive to cytotoxic chemotherapy than midgut NETs. Until recently, randomized clinical trial (RCT) data comparing chemotherapy regimens for pNETs was lacking;^18^ however, a recent randomized phase II trial showed that the combination temozolomide and capecitabine was associated with a longer progression-free survival (PFS) than temozolomide alone.^19^

Discrete choice experiments (DCE) are a quantitative method for eliciting preferences. Participants are asked to choose between a series of options within scenarios comprised of several attributes, whose values or levels are varied between the choices. Analysis of the pattern of choices is used to construct a model of the relative importance or utility of each attribute to participant decision making. There is growing interest in the use of DCEs in the healthcare setting, with studies focusing on topics from patient-level clinical decision making through to health policy design and implementation.^20^ DCE methodology has previously been applied to modeling treatment decisions in oncology,^5-7,21-25^ however there have been no studies to date on treatment preferences for NET patients. Treatment options available for advanced, unresectable NETs vary greatly in terms of their attributes, including side effect profiles, frequency and methods of administration, and associated progression-free survival. There are clinical scenarios with equipoise between treatments based on survey of expert clinicians.^10^ We, therefore, investigated the relative importance of treatment attributes to NET patients using DCEs based on available randomized control trial data for NET treatments to better understand patient preferences and priorities around treatment.

### Methods

#### Study Objective and Outcomes

The primary objective of the study was to use discrete choice experiments to elicit patient preferences with respect to the treatment attributes (progression-free survival (PFS), adverse event rates, and method of administration), and profiles of attributes representing specific existing treatment options, for advanced NETs.

The primary outcome was attribute rankings based on utility values representing patient preferences for treatment attributes, including progression-free survival, treatment modality, and rates of adverse events. The secondary outcome was ranking of attribute profiles matching specific treatments for advanced NETs.

#### Patient Population and Recruitment

Participants were eligible for the study if they had been diagnosed with a NET. The DCEs were implemented as anonymous online surveys. Participants self-enrolled after agreeing to the online consent form, and adherence to eligibility criteria and collected demographic data was based on self-report. The study website linked to 2 different DCE surveys as described below. Consenting participants were randomized to complete one of the 2 surveys using Javascript solely to balance the number of participants between them. The study was organized under the auspices of the Commonwealth Neuroendocrine Tumor Collaboration (CommNETs). Online social media and email recruitment were assisted by NET patient advocacy groups, including: Carcinoid Cancer Foundation, NeuroEndocrine Cancer New Zealand, NeuroEndocrine Cancer Australia, Canadian Neuroendocrine Tumor Society, and the International Neuroendocrine Cancer Alliance. Recruitment occurred between September 24, 2020 and July 8, 2021. Ethical approval for this study was granted by the Sunnybrook Research Ethics Board (ID:2301) and University of Otago Human Ethics Committee (Health) (D20/429).

#### Discrete Choice Experiment Development

A DCE is a method for modeling the underlying preferences that determine an individual’s choice between a given set of options. Participants are asked to make choices between hypothetical treatments that vary based on several attributes, and the trade-offs they make are used to determine the relative importance of the attributes to their choices. In keeping with published best practices,^26^ development of the DCEs presented involved several sequential steps. Provisional DCE scenarios and content (attributes to model) were initially selected based on literature and guideline review by the investigators. Proposals for the DCEs were reviewed at CommNETs meetings, attended by NET healthcare providers, NET patient advocacy groups, and NET patients.^27^ A pilot study of the draft surveys was then conducted with 5 NET patients recruited from investigators’ clinical practices, who subsequently completed a telephone interview about the surveys with a study investigator. The aim of the pilot was both to determine content validity of the surveys, and to screen for any technical difficulties with the online survey tool. Revisions to the study website and survey language were made based on the interviews, and the DCE surveys finalized. Given changes to the survey structures, the results from the 5 participants who completed the pilot were not included in the final dataset.

The DCEs were designed to model 2 scenarios faced by NET patients and clinicians where there are several treatment options, with no dominant first-line choice. They were: (1) second-line treatment for midgut NETs that have progressed on SSAs; (2) second-line treatment for pNETs that have progressed on SSAs. Levels for these attributes were obtained directly from the randomized clinical trial data supporting their use in the relevant NET population. Treatments included for scenario 1 are: ^177^Lu-Dotatate,^15^ somatostatin analogue dose escalation,^15^ and everolimus.^14^ Treatments included for scenario 2 are: sunitinib,^17^ everolimus,^16^ and capecitabine/temozolomide (CAPTEM).^19^ The modeled treatments and their associated attributes and levels are shown in Table 1. Adverse event (AE) rates presented represent the frequency of grade 3 or higher AEs in the relevant RCT. The participant facing language employed on the online DCE survey is available in Supplementary Tables S1 and 2. As participants were assigned randomly to either DCE irrespective of their self-reported NET diagnosis, participants with a history of pNET could complete either the DCE based on pNET or midgut NET clinical trial data, and vice versa.

**Table 1.** Discrete choice experiment scenarios, attributes, and levels.

Midgut NET Scenario					
Treatment profile	Treatment attributes				
	PFS	Modality	Diarrhea	Mucositis	Secondary leukemia
PRRT	29 months	Intravenous (IV) treatment in the hospital every 2 months, for a total of 4 treatments. One intramuscular (IM) injection each month^*^.	3 out of 100 patients	0 out of 100 patients	1 out of 100 patients
Everolimus	11 months	One pill each day	7 out of 100 patients	9 out of 100 patients	0 out of 100 patients
SSA dose escalation	8 months	One IM injection every 2 weeks	2 out of 100 patients	0 out of 100 patients	0 out of 100 patients

PNET scenario					
	Treatment attributes				
Treatment profile	PFS	Modality	Mucositis	Hand-foot syndrome	Infections/febrile neutropenia
CAPTEM	23 months	Two different pills for 2 weeks, then 2 weeks’ break.	0 out of 100 patients	0 out of 100 patients	13 out of 100 patients
Sunitinib	11 months	One pill each day	4 out of 100 patients	6 out of 100 patients	12 out of 100 patients
Everolimus	11 months	One pill each day	7 out of 100 patients	0 out of 100 patients	2 out of 100 patients

^*^As per the treatment protocol in the NETTER-1 trial,^15^ patients continued to receive IM injections of SSA while undergoing PRRT.

#### 1000minds Conjoint Analysis

The DCEs employed the “potentially all pairwise rankings of all possible alternatives” (PAPRIKA) method,^28^ as implemented by the conjoint analysis workflow in the 1000minds software package.^29^ PAPRIKA allows for the presentation of simple choices between 2 attribute partial profiles in the form of an adaptive questionnaire. An example choice question is shown in Supplementary Fig. S1. The number of choices required to be made between partial profiles is minimized by using the property of transitivity to implicitly identify pairs of hypothetical alternatives that can be ranked based on previous choices, and then eliminating those from the choices that will be shown to participants. The simple partial-profile choices presented, and the reduced number of choices required, using this method theoretically reduce choice fatigue. The method outputs part-worth utility values for each attribute level for individual participants, in contrast to other DCE methods that produce only aggregate data. The part-worth utility values for attribute levels can be used to rank the relative influence of different attributes on each participant’s decision making. Likewise, the part-worth utility values for the combination of attribute levels that match each of the modeled real-world treatment options shown in Table 1 can be used to rank each participant’s preference for the different treatment profiles. Participant-level rank values were summarized with descriptive statistics.

## Results

The study population is described in Table 2. Participants who completed the midgut NET scenario (*N* = 110) had a median age of 61.5, and were predominantly female (68.2%), located in North America (59.1%), had gastrointestinal tract (59.1%) or pNETs (14.5%), and had metastatic disease (78.2%). Participants who completed the pNET scenario (N=132) had a median age of 63.0, and were likewise predominantly female (68.2%), located in North America (61.4%), had gastrointestinal tract (61.4%) or pNETs (28.0%), and metastatic disease (90.9%).

**Table 2.** Study populations.

	Midgut scenario (*N* = 110)	PNET scenario (*N* = 132)
Age (median, SD)	61.5 (12.4)	63.0 (10.9)
Gender		
Female	75 (68.2%)	90 (68.2%)
Male	34 (30.9%)	41 (31.1%)
No response	1 (0.9%)	1 (0.7%)
Participant location		
North America	65 (59.1%)	81 (61.4%)
Europe	7 (6.4%)	10 (7.6%)
Oceania	36 (32.7%)	37 (28.0%)
Africa	0 (0.0%)	1 (0.8%)
Asia	1 (0.9%)	3 (2.3%)
Primary tumor		
GI	65 (59.1%)	74 (56.1%)
Lung	15 (13.6%)	17 (12.9%)
Pancreas	16 (14.5%)	32 (24.2%)
Other	14 (12.7%)	9 (6.8%)
Extent of disease		
Localized	21 (19.1%)	12 (9.1%)
Metastatic/locally advanced	86 (78.2%)	120 (90.9%)
Couldn’t recall	3 (2.7%)	0 (0.0%)

**Figure 1.**
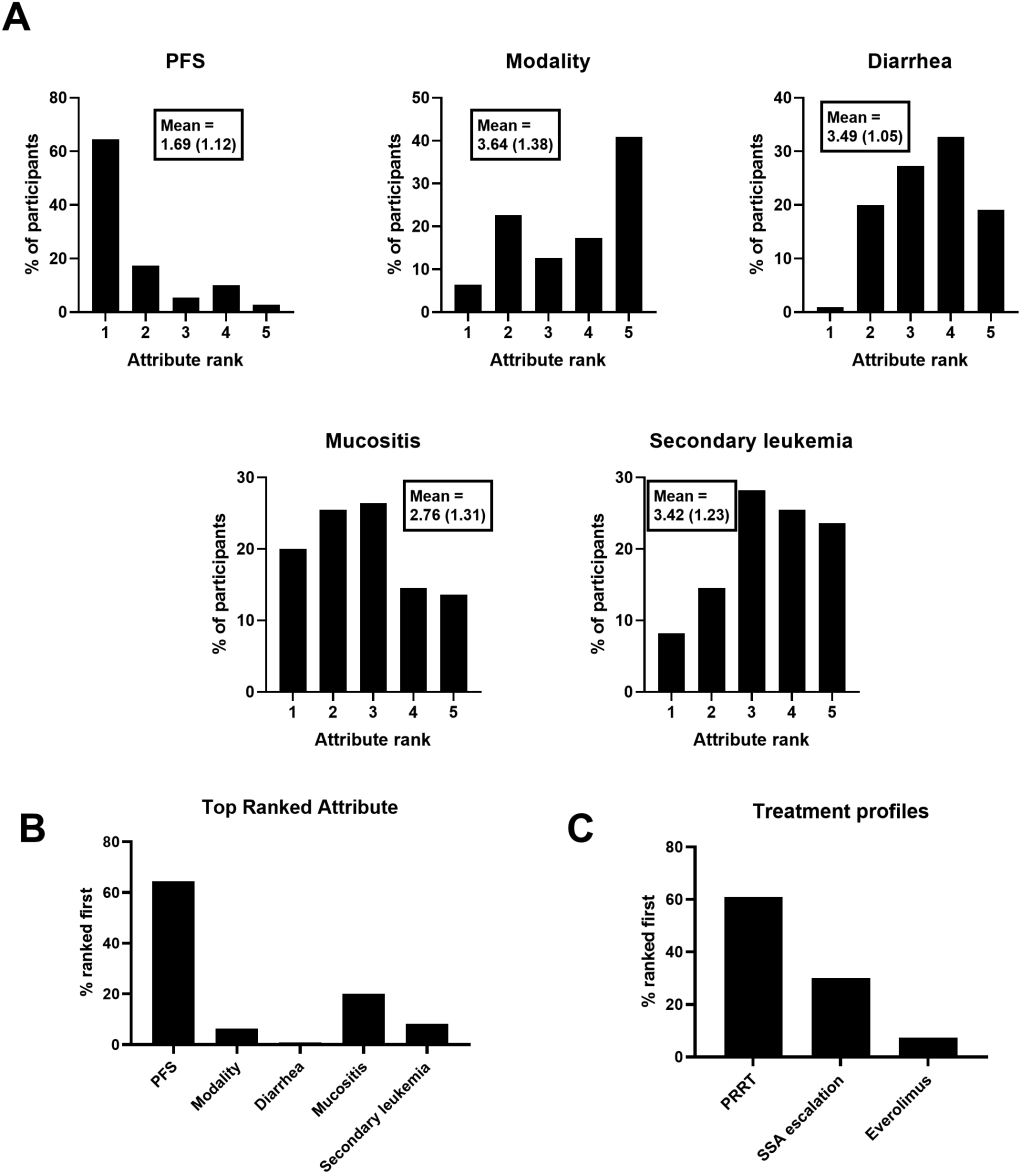
Midgut NET scenario results. Ranks are derived from individual participant part-worth utility values for each attribute level. (**A**) Distribution of attribute ranks among participants (Mean rank and standard deviation shown in boxes). (**B**) Proportion of participants where each attribute was ranked first. (**C**) Proportion of participants where each treatment profile was ranked first.

Results from the midgut NET scenario are shown in Fig. 1. Each participant’s individual part-worth utility values for the different attribute levels were used to rank the attributes, indicating the relative contribution or importance to their decision making during the DCE scenario. A higher rank therefore indicates that the treatment attribute was judged to be more important during the decision making process. Longer PFS had the highest mean rank, follow by reduced occurrence of mucositis, secondary leukemia, and diarrhea in descending order (Fig. 1A). Treatment modality had the lowest mean rank, with the ranking within the attribute descending as follows: oral therapy, IM injection, IV + IM. When the data are represented as the proportion of participants’ where an attribute was ranked first in terms of influence on decision making (Fig. 1B), PFS was most frequently ranked first (64.5% of participants), followed by reduced frequency of mucositis (20.0%), reduced frequency of secondary leukemia (8.2%), treatment modality (6.4%), and reduced frequency of diarrhea (0.9%). The part-worth utility values for the attribute levels matching specific treatment profiles also allows for ranking each participants preference for those treatment profiles. 60.9% of participant responses matched a preference for PRRT, 30.0% for SSA dose escalation, and 7.3% for everolimus (Fig. 1C). Two participants (1.8%) gave responses resulting in equal part-worth utility values for the attribute levels matching PRRT and SSA dose escalation (data not shown in Fig. 1C).

**Figure 2.**
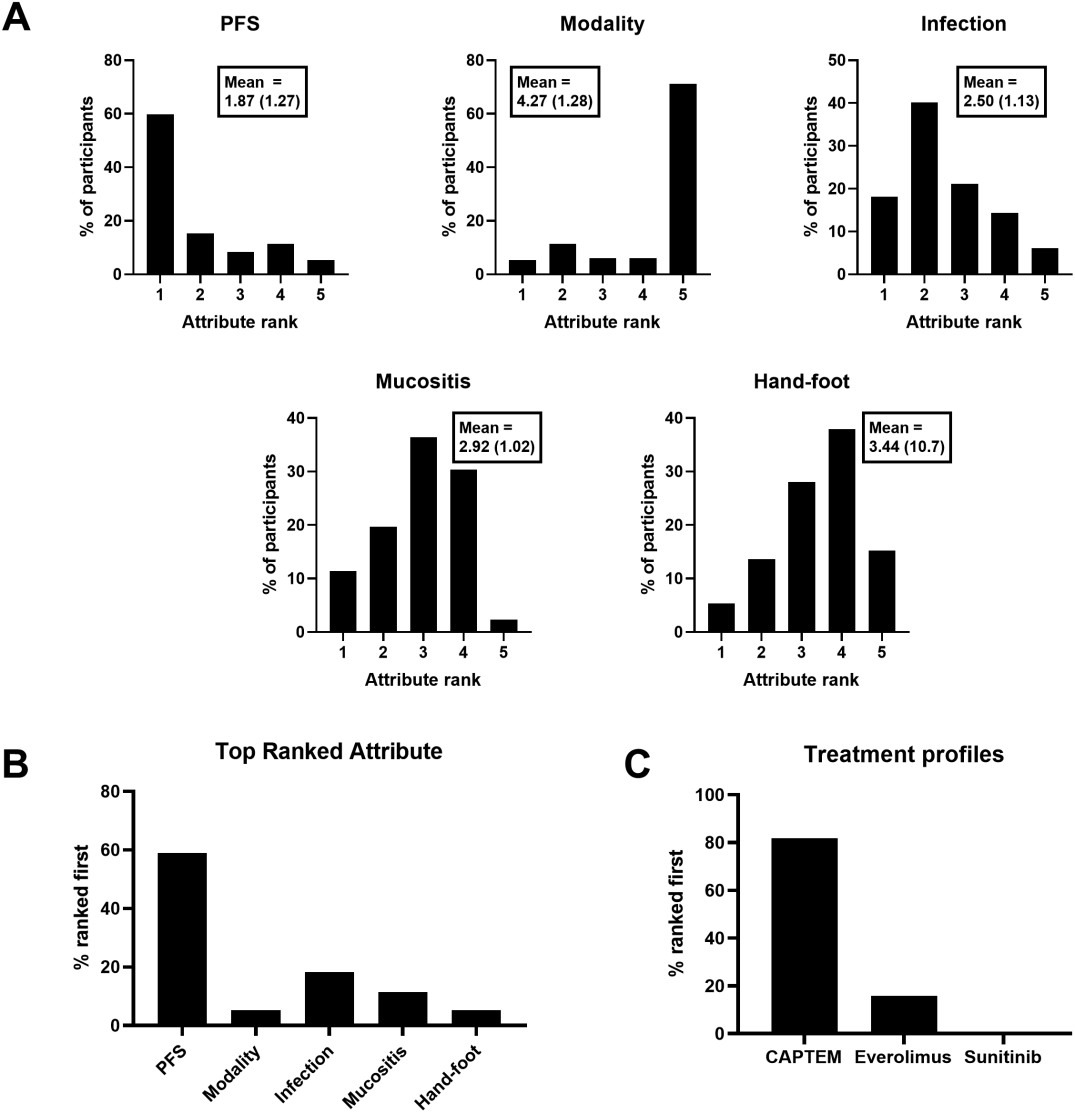
PNET scenario results. Ranks are derived from individual participant part-worth utility values for each attribute level. (**A**) Distribution of attribute ranks among participants (Mean rank and standard deviation shown in boxes). (**B**) Proportion of participants where each attribute was ranked first. (**C**) Proportion of participants where each treatment profile was ranked first.

Results from the pNET scenario are shown in Fig. 2. Longer PFS had the highest mean rank, followed by reduced occurrence of infection, mucositis, and hand-foot syndrome in descending order (Fig. 2A). Treatment modality had the lowest mean rank, with one daily pill (everolimus, sunitinib) ranked higher than the 2 pill chemotherapy cycles (CAPTEM). When the data are represented as the proportion of participants’ where an attribute was ranked first in terms of influence on decision making (Fig. 2B), PFS was most frequently ranked first (59.0% of participants), followed by reduced frequency of infection (18.2%), and reduced frequency of mucositis (11.4%). Treatment modality and reduced frequency of hand-foot syndrome were both ranked first in 5.3% of participants. Ranking of attribute profiles matching specific treatments identified a preference for CAPTEM in 81.8% of participants, 15.9% for everolimus, and 0.0% for sunitinib (Fig. 2C). One participant (0.8%) gave responses resulting in equal part-worth utility values for the attribute levels matching CAPTEM and everolimus, and 2 participants (1.5%) gave responses resulting in equal part-worth utility values for the attribute levels matching all 3 treatment profiles (data not shown in Fig. 2C).

## Discussion

Patients with advanced NETs have a variety of potential treatment options following progression on SSAs. Shared decision making, a key aspect of patient-centered cancer care, requires an understanding of and attention to individual patient preferences and values. Patients and oncologists have been shown to place different value on important treatment attributes such as risk of adverse events when assessed in isolation.^5-7^ Providing oncologists with high-quality data on patient preferences has the potential to help better align treatment decisions with patient values. Here, we have presented results from 2 DCEs, where patient participants made choices between hypothetical treatments whose attributes were based directly on the RCT data that underlies the use of second-line therapies for advanced midgut NETs and pNETs. The DCE methodology used here allowed both for the presentation of data from the entire study population, but also for individual participants, revealing clear heterogeneity in decision-making priorities.

We found that longer PFS, the accepted primary end in NET trials due to their long overall survival, was the treatment attribute to which patient participants attributed the most importance in their decision making for approximately 60% of individuals in each scenario. Conversely, this means that approximately 40% of participants placed more value on other treatment attributes, such as lower frequency of severe adverse events. Treatment modality was the attribute with the lowest mean rank in each scenario. Despite this, there were still 5% of patients in the midgut scenario and 6% in the pancreatic scenario who rated treatment modality as the most important priority. These results highlight considerable heterogeneity in patient priorities concerning treatment options in the context of these DCE models, in which the attribute levels were directly derived from the RCTs supporting the use of these medications in clinical practice. Similar conclusions can be drawn from the participant-level data of utility values matching specific treatment profiles. In the midgut NET scenario the responses of 60.9% of participants suggest a preference for PRRT, however, a sizeable minority of 30.0% made responses in keeping with preference for an attribute profile matching SSA dose escalation. While the pNET scenario showed a more clearly preferred treatment in CAPTEM in 81.8% of participants, still 15.9% of participants made choices suggesting preference for the everolimus attribute profile, despite the markedly lower associated PFS.

DCE studies of treatment decisions in other oncology settings have identified longer progression-free survival as the treatment attribute most important to patients, in comparison to AE rates or treatment modality.^6,23,24^ However, a DCE study of castration-resistant prostate cancer in Japan identified reduction of tumor-related symptoms and avoidance of treatment side effects as more important than overall survival.^22^ Multiple studies have shown differences in preferences between patients and oncologists.^5-7^ These DCE studies also serve to highlight the diversity treatment preferences among oncology patients, which may vary based on tumor type, stage, and individual patient and cultural factors.

Shared decision making is an expected standard in modern cancer care, as reflected in the publication of guidelines focused on patient-centered care.^30,31^ However, some studies have shown that the proportion of oncology patients who feel like they were offered a treatment choice or asked about their preferences can be as low as 50%.^32,33^ Even when physicians are motivated to engage in shared decision making, the process can be impacted by a variety of cognitive biases, influenced by how and what information is presented by the clinician, and how information is received and assessed by the patient and caregivers.^8^ Given the inequitable access to relevant information at the time of decision making, physicians likely have the greater influence even on shared decisions.^9^ Effective shared decision making may, therefore, benefit from both an understanding of the clinician’s own biases, but also what information might be important to a patient facing the given decision. The results of the current study highlight significant heterogeneity in preferences and priorities for treatment attributes and treatment profiles among participants. These results therefore can serve as a reminder to clinicians to engage their NET patients in detailed discussions about treatment choices that include relevant tradeoffs such as those between efficacy and adverse event rates. A further implication is the relative importance of adverse events and quality of life factors including method of administration on decision making. Reports of clinical trial results typically emphasize survival outcomes; however, complete reporting of adverse event occurrence and severity, and quality of life data, may be just as important in helping patients and clinicians make treatment decisions.

Discrete choice experiments have several advantages over patient preference surveys. In surveys, patients are often asked to rate how important certain features are, such as control of tumor growth or reduction in symptoms. Most survey methods allow both to be rated highly, whereas the discrete choice experiment requires that participants choose between outcomes. Our research has demonstrated that there is no one endpoint that can be characterized as most important to patients. This has several implications. First, clinical trials usually select a single measure as the primary outcome variable. Our findings show that while one outcome measure is important to a proportion of patients, several others would prefer differing endpoints. Regulatory approvals are also frequently granted on the basis of PFS. Our study indicates that patients do not universally value control of tumor growth over symptom control. We consider that these important findings should support ongoing efforts to ensure that patient preferences inform trial design such that patient-centered endpoints are consistently measured and reported. This would lead to study designs that reported results that reflected the variety of values that are held by a patient population rather than a sole focus on radiological endpoints.

This study has several important strengths. The DCEs were based on the RCT data used to support the currently available treatment options for advanced NETs. This included modeling treatments from 2 different clinical scenarios, providing the opportunity to compare between them. Relative to other DCEs modeling oncology treatment decisions, the methodology employed in this study provides patient level data, allow for a more direct assessment of the heterogeneity between individual participants. The study benefits from the general strength of DCEs, namely the production of quantitative data on explicit preferences. Limitations include a relatively modest sample size for each DCE, and that only a limited set of attributes can be modeled in a DCE, which do not represent a full characterization of a cancer therapy. However, previous work has identified strong correlation between choices made on a DCE and in equivalent real-world scenarios.^34,35^

## Conclusion

In summary, our study suggests that NET patients have heterogeneous priorities when choosing between treatment options based on the results of 2 independent DCEs. These results highlight that knowledge of patient preferences should be incorporated into trial endpoints, trial design, and regulatory considerations in order to drive research that focuses on delivering outcomes of value to a diverse patient population.

## Supplementary Material

oyad312_suppl_Supplementary_Material

## Data Availability

The data underlying this article will be shared on reasonable request to the corresponding author.
