# Supplementary material for: Patient Priorities Concerning Treatment Decisions for Advanced Neuroendocrine Tumors Identified by Discrete Choice Experiments
Source: Oncologist. 2023 Nov 25;29(3):227–34. doi: 10.1093/oncolo/oyad312 (PMC10911922; doi:10.1093/oncolo/oyad312)
Supplement: oyad312_suppl_Supplementary_Material [file oyad312_suppl_supplementary_material.docx]

Supplementary Table 1: Attributes and levels derived from clinical trials for three treatment options for patients with advanced midgut NETs that have progressed after 1^st^ line treatment with somatostatin analogues

|  | ^177^Lu-Dotatate | Somatostatin analogue dose escalation | Everolimus |
| --- | --- | --- | --- |
| Average length of time before tumour begins to grow again | 29 months | 8 months | 11 months |
| How treatment is given | An intravenous (IV) treatment in the hospital every 2 months, for a total of 4 treatments. You will also have one needle each month. | One needle every 2 weeks | One pill each day |
| Chance of having diarrhea so badly that you had to go to the hospital | 3 out of 100 patients | 2 out of 100 patients | 7 out of 100 patients |
| Chance of having painful mouth or lip sores that make it hard to eat or drink | 0 out of 100 patients | 0 out of 100 patients | 9 out of 100 patients |
| Chance that you may eventually develop a blood cancer as a result of the treatment | 1 out of 100 patients | 0 out of 100 patients | 0 out of 100 patients |

Supplementary Table 2: Attributes and levels derived from clinical trials for three treatment options for patients with advanced pancreatic NETs previously treated with lanreotide

|  | Everolimus | CAPTEM | Sunitinib |
| --- | --- | --- | --- |
| Average length of time before tumour begins to grow again | 11 months | 23 months | 11 months |
| How treatment is given | One pill each day | Two different pills, taken for two weeks out of every four weeks | One pill each day |
| Chance of having painful mouth or lip sores that make it hard to eat or drink | 7 out of 100 patients | 0 out of 100 patients | 4 out of 100 patients |
| Chance of having painful reddening, swelling, or numbness on palms of the hands and soles of the feet | 0 out of 100 patients | 0 out of 100 patients | 6 out of 100 patients |
| Chance of having a serious infection where you needed to be treated in a hospital | 2 out of 100 patients | 13 out of 100 patients | 12 out of 100 patients |


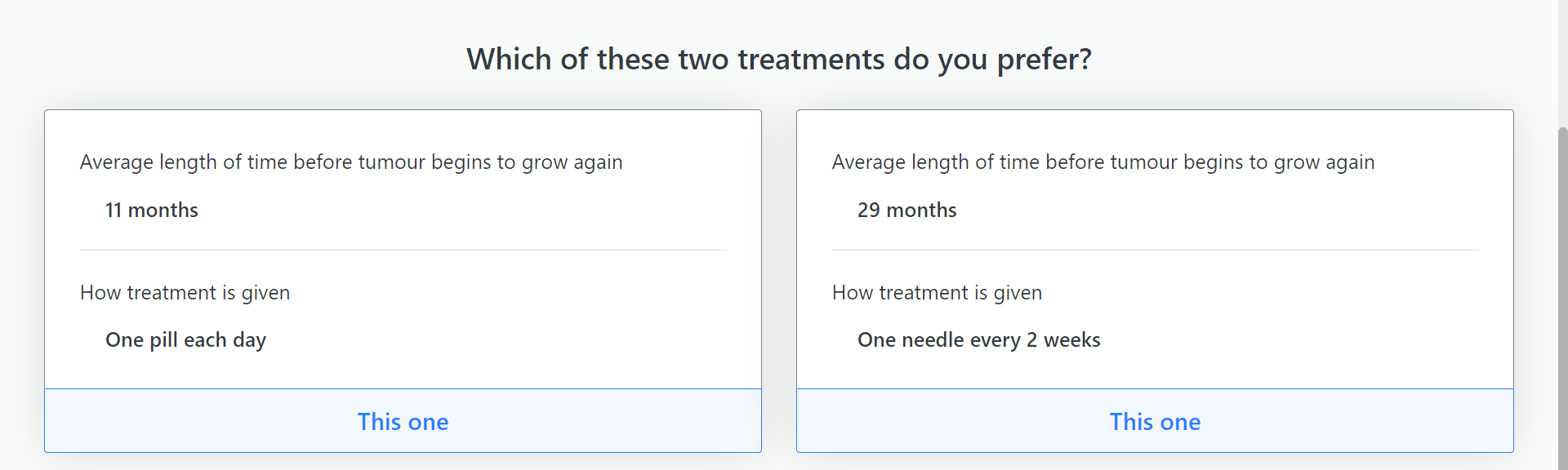

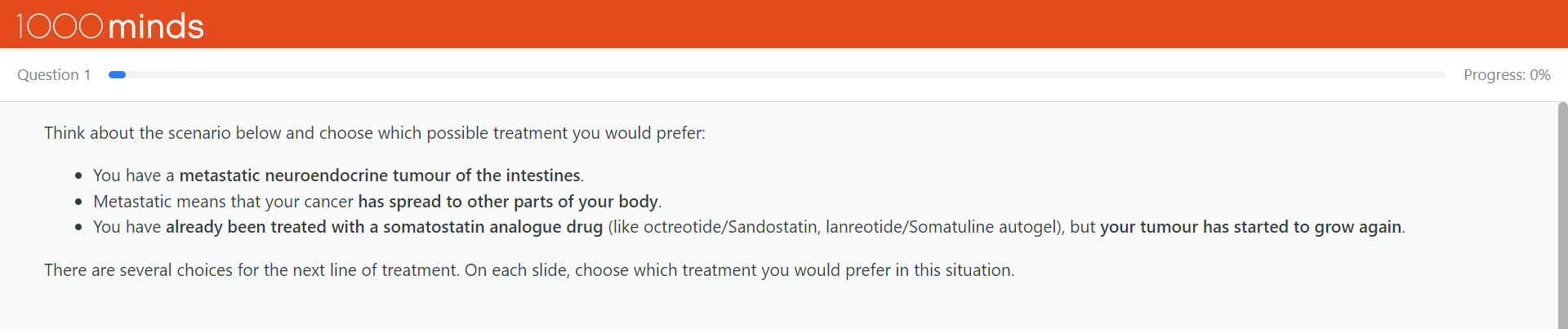


Supplementary Figure 1: Representative choice question presented during adaptive DCE on the 1000minds platform.
